# Genome-wide phylogenetic analysis of the pathogenic potential of *Vibrio furnissii*

**DOI:** 10.3389/fmicb.2014.00435

**Published:** 2014-08-21

**Authors:** Thomas M. Lux, Rob Lee, John Love

**Affiliations:** Biosciences, College of Life and Environmental Sciences, The University of ExeterExeter, UK

**Keywords:** *Vibrio furnissii*, horizontal gene transfer, genome comparison, emerging pathogens, pathogenicity islands, phylogenetic analysis, genome phylogeny

## Abstract

We recently reported the genome sequence of a free-living strain of *Vibrio furnissii* (NCTC 11218) harvested from an estuarine environment. *V. furnissii* is a widespread, free-living proteobacterium and emerging pathogen that can cause acute gastroenteritis in humans and lethal zoonoses in aquatic invertebrates, including farmed crustaceans and molluscs. Here we present the analyses to assess the potential pathogenic impact of *V. furnissii*. We compared the complete genome of *V. furnissii* with 8 other emerging and pathogenic *Vibrio* species. We selected and analyzed more deeply 10 genomic regions based upon unique or common features, and used 3 of these regions to construct a phylogenetic tree. Thus, we positioned *V. furnissii* more accurately than before and revealed a closer relationship between *V. furnissii* and *V. cholerae* than previously thought. However, *V. furnissii* lacks several important features normally associated with virulence in the human pathogens *V. cholera* and *V. vulnificus*. A striking feature of the *V. furnissii* genome is the hugely increased Super Integron, compared to the other *Vibrio*. Analyses of predicted genomic islands resulted in the discovery of a protein sequence that is present only in *Vibrio* associated with diseases in aquatic animals. We also discovered evidence of high levels horizontal gene transfer in *V. furnissii*. *V. furnissii* seems therefore to have a dynamic and fluid genome that could quickly adapt to environmental perturbation or increase its pathogenicity. Taken together, these analyses confirm the potential of *V. furnissii* as an emerging marine and possible human pathogen, especially in the developing, tropical, coastal regions that are most at risk from climate change.

## Introduction

The Vibrionales are a diverse order of free-living, gram-negative proteobacteria, found throughout the world in marine and freshwater environments (Baumann et al., 1983; Thompson et al., 2004). *Vibrio cholerae*, *V. parahaemolyticus* and *V. vulnificus* are potentially lethal human pathogens (Baumann et al., 1983; Chakraborty et al., 1997; Rivera et al., 2001; Colwell, 2004; Thompson et al., 2004) and at least eight other *Vibrio* species pose a serious threat to human health either through ingestion of contaminated food—notably raw seafood—or by exposure of skin lesions to aquatic environments and marine animals. These “emerging *Vibrio*” include *Vibrio furnissii*, a widespread, marine species (Hickman-Brenner et al., 1984). *V. furnissii* is considered a relatively weak human pathogen, although is has been implicated in occasional outbreaks of acute gastroenteritis in which deaths have been recorded (Chakraborty et al., 1997; Dalsgaard et al., 1997; Wu et al., 2007). More importantly, *V. furnissii* has caused zoonoses in marine invertebrates (Matté et al., 1994; Austin, 2010) as significant as those caused by other species such as *V. corallyticus* (Ben-Haim et al., 2003) and *V. splendidus* (Gay et al., 2004). It is therefore possible that *V. furnissii* could present an increasing risk to human economic activity, notably the production of farmed shellfish in developing countries.

The *V. furnissii* genome (Strain NCTC 11218) (Hickman-Brenner et al., 1984) was completely sequenced, assembled and annotated (Lux et al., 2011). In the present analysis, we have assessed the pathogenic potential of *Vibrio furnissii*. Individual phylogenetic trees for all predicted gene products were constructed and showed a closer evolutionary relationship between *V. furnissii* and *V. cholerae* than hitherto recognized. An identification of putative pathogenicity islands in 8 sequenced *Vibrionaceae* genomes and analysis of their distribution revealed stark congruities. Moreover, we noted a high abundance of horizontal gene transfer in the *V. furnissii* genome from other marine bacteria. Taken together, these analyses confirm the potential of *V. furnissii* as an emerging marine and possible human pathogen.

## Materials and methods

### Representation and analysis of the *Vibrio furnissii* genome

We performed *in silico* analyses of all predicted protein sequences. Gene products were scanned for clusters of orthologous groups (COG) and were mapped to COG categories using AutoFACT software (Tatusov et al., 2000; Koski et al., 2005). The possible functions of each translated polypeptide were determined by comparing the *V. furnissii* proteome to the Pfam-A database (Bateman et al., 2000; Finn et al., 2006) and to the Kyoto Encyclopedia of Genes and Genomes (KEGG) database (Kanehisa et al., 2002). Transmembrane helices (TMH) were predicted using Phobius software (Kall et al., 2004, 2007) and the genome was scanned for tRNAs and tmRNAs using tRNAscan-SE 1.23 and ARAGON software respectively. RNAmmer scanned for rRNAs (Lowe and Eddy, 1997; Laslett and Canback, 2004; Lagesen et al., 2007). Results from the *in silico* proteome analyses were assessed relative to each annotated gene and appropriate comments incorporated into the published genome annotation.

The assembled genome and follow-up analyses results were represented using circos software (Krzywinski et al., 2009).

### Whole genome alignments with other *Vibrionaceae*

The *V. furnissii* genome was aligned against 8 *Vibrionaceae* genomes representing a selection of human, fish and invertebrate pathogens (i.e., the “emerging *Vibrio*”), as well as the accepted pathogens *V. cholerae* and *V. vulnificus*, using MUMmer 3.0 software (Delcher et al., 2002). *V. furnissii* was used as the reference and independently compared to the genomes of *V. cholerae* O1 biovar El Tor strain N16961 (NC002505 and NC002506), *V. cholerae* O395 (NC009457 and NC009456), *V. parahaemolyticus* strain RIMD 2210633 (NC004603 and NC004605), *V. vulnificus* strain CMCP6 (NC004459 and NC004460), *V. harveyi* strain ATCC BAA-1116 (NC009783, NC009784 and NC009777) and *V. splendidus* strain LGP32 (FM954972 and FM954973). The genomes of *Listonella anguillarum* (formerly *Vibrio anguillarum*) 775 (NC015633 and NC015637) and *Photobacterium profundum* strain SS9 (NC006370 and NC006371), both members of the Vibrionales were used as outliers. The settings for MUMmer were defined as maxgap = 500 and mincluster = 100.

### Phylogenetic analysis

The phylogeny of each predicted gene product encoded in the *V. furnissii* NCTC 11218 genome was determined using a previously described Perl script that we adapted for use with bacterial genomes (Richards et al., 2009). The script uses the Basic Local Alignment Search Tool (BLAST; *e*-value 1e^−20^) to identify local similarities between the amino acid sequence of each CDS and a database containing the protein sequences of 680 bacterial genomes. The amino acid sequences corresponding to the top BLAST hits (range 3–5000) were retrieved from the database and alignments were performed using MUSCLE software. Conserved positions were located with Gblocks software (Talavera and Castresana, 2007) and PhyML software running an approximate likelihood-ratio test (aLRT) algorithm was used to rapidly construct the phylogenetic trees (Guindon and Gascuel, 2003; Anisimova and Gascuel, 2006) using the parameters described in Richards et al. (2009). The phylogenetic trees of all *V. furnissii* proteins in Newick format are available as supplemental material.

**Figure 1:**
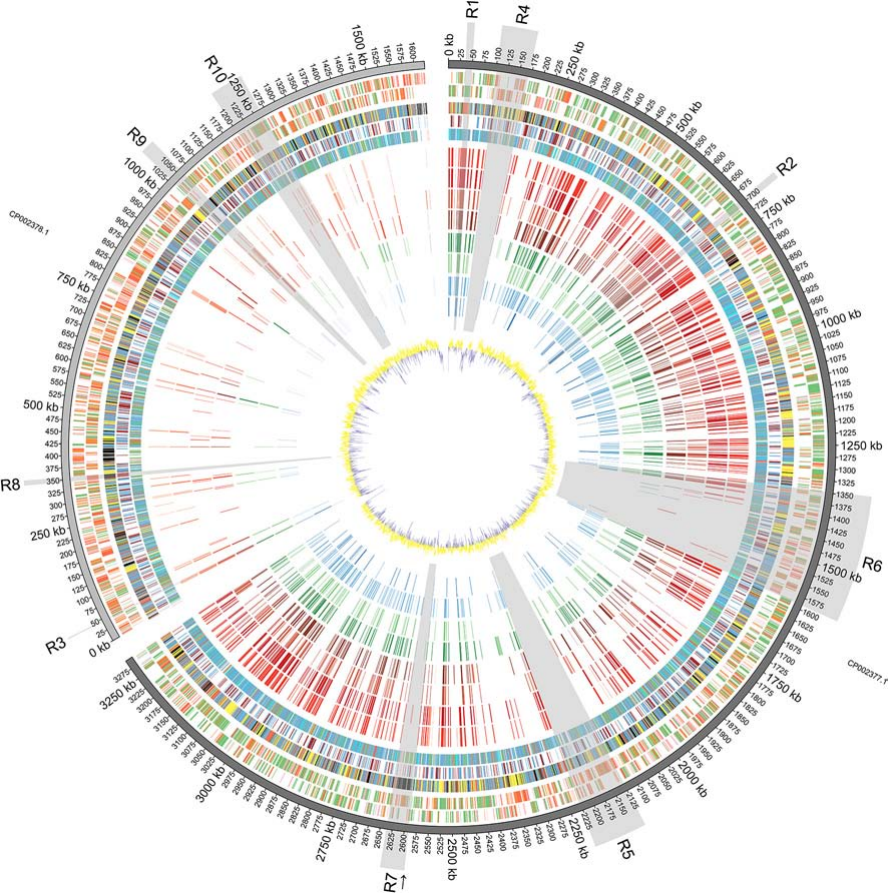
**Diagrammatic Representation of the *Vibrio furnissii* Genome:** From outside to inside: Predicted ORFs on *V. furnissii* forward strand (track 1) and reverse strand (track 2). Green represents 100–80% amino acid identity; Orange, 80–60% identity and Red below 60% identity; COG classification (track 3; Information storage and processing: pink, Cellular processes and signaling: yellow, Metabolism: blue, Poorly characterized: Gray, No hits: black), TMH and SP prediction (track 4; Blue: THM, Light green: SP; Brown: THM+SP), representation of phylogenetic trees (track 5; Red: 3–10 species per leaf; Green: 11–100 species per leaf; Blue: 101–1000 species per leaf; Cyan: 1001 to END species per leaf), NUCMER comparison of 8 bacteria: *V. cholerae* O1 biovar El Tor strain N16961 (track 6), *V. cholerae* O395 (track 7), *V. parahaemolyticus* strain RIMD 2210633 (track 8), *V. vulnificus* strain CMCP6 (track 9), *V. harveyi* strain ATCC BAA-1116 (track 10), *V. splendidus* strain LGP32 (track 11), Listonella (Vibrio) anguillarum 775 (track 12) and Photobacterium profundum strain SS9 (track 13), against *V. furnissii* (regions sharing more than 80% identity are illustrated by bars; red hues represent human associated, green hues represent invertebrate and fish associated, and blue hues represent fish associated pathogens; darker color for identical regions on chromosome I, lighter color for chromosome II), GC plot (track 14; Window size: 10,000, Step size 200).

Phylogenetic trees were build using either 16s rRNA nucleotide sequences or nucleotide sequences of the genomic regions of interest R1, R2 and R3, as indicated on Figure 1. This was performed using the programs describe above.

To help establish an even more detailed phylogenetic analysis between *V. furnissii* and the 8 selected *Vibrio*, the core genome (genes shared by all the strains) and the pangenome (the core genome combined with strain-specific and partially shared genes) were constructed (Tettelin et al., 2008), using a program developed and described by Contreras-Moreira and Vinuesa (2013). In an initial step, the genbank files for each chromosome of each *Vibrio* species were combined to include the genes of both chromosomes. The second step involved building the core genome and pangenome with default values using the bidirectional best-hit algorithm. In the final step a phylogenetic tree using the provided script was constructed.

### Determination of genomic islands

Genomic islands in the genomes of *V. furnissii* and of the 8 emerging *Vibrio* were identified using IslandViewer (Waack et al., 2006; Langille et al., 2008; Langille and Brinkman, 2009). Polypeptide sequences within the identified Genomic Islands of *V. furnissii* were compared against all proteins of the 8, selected *Vibrio* by BLASTP, and parsed using a 30% or higher identity filter.

## Results

### The *Vibrio furnissii* genome

The assembled *V. furnissii* genome is accessible through the NCBI under accession numbers CP002377 (chromosome I) and CP002378 (chromosome II). General features are described in Lux et al., (2011).

The *V. furnissii* genome was aligned against a selection of 8 fully sequenced *Vibrio* genomes representing the pathogenic *Vibrio* and 1 outlier. Conserved regions of at least 100 nucleotides in length with at least 80% nucleotide homology between the *V. furnissii* sequence and each of the compared bacterial species were highlighted, and occur predominantly on chromosome I (Figure 1, track 6–13).

Chromosome I contains 1058 complete CDS with at least 80% nucleotide homology to other members of the sequenced *Vibrionaceae*, representing 33.53% of the total number of CDS located on chromosome I. Conversely, chromosome II contains only 152 complete, homologous CDS, which is 9.87% (with over 80% nucleotide homology) of all genes located on chromosome II. Most homology occurred within chromosomes, but in some cases individual CDS appear to have swapped chromosomes; for example, the gene encoding Threonyl-tRNA synthetase (VfuB00041; 44369–46297) is located on chromosome II in the genomes of *V. furnissii*, *V. cholerae* and *P. profundum*, but is situated on chromosome I of *V. vulnificus*, *V. parahaemolyticus*, *V. harveyi*, *V. splendidus* and *V. fischeri* (Figure 1, R7). Conversely, the genes encoding tRNAs for glutamine, lysine and valine are located on chromosome II of *V. harveyi*, but appear on chromosome I in all the other sequenced *Vibrio* (Figure 1, track 6–13).

### Genomic comparisons between the *Vibrionaceae*

The analyses of nucleotide homology between the sequenced *Vibrio* enabled us to select 10 regions of particular interest (shaded areas in Figure 1). That are either present in all *Vibrio* strains or present only in *V. furnissii*.

### Regions present in all selected *Vibrio*

Regions R1 (35,611 bp—49,550 bp; VfuA00037-VfuA00045) and R2 (692,714 bp—705,975 bp; VfuA00666-VfuA00691) are highly conserved between all the compared sequences. All CDS in either region are present on chromosome I of the compared sequences and fall into the “Information Storage and Processing” COG category. More specifically, region R1 comprises 4 genes that encode tRNAs for threonine, glycine, tyrosine and threonine (VfuAtRNA10–VfuAtRNA13) and genes encoding a DNA-directed RNA polymerase (VfuA00037), 50S ribosomal proteins (VfuA00039–VfuA00042), an elongation factor Tu (VfuA00045), a preprotein translocase (SecE) subunit (VfuA00044) and a transcription anti-terminator (VfuA00043). The encoded peptides have no predicted trans-membrane helices or signal peptide sequences.

Region R2 includes 14 genes that encode 50S ribosomal proteins (VfuA00667–VfuA00670, VfuA00672, VfuA00677–VfuA00679, VfuA00682, VfuA00683, VfuA00685, VfuA00686 and VfuA00688) and 8 genes encoding 30S ribosomal proteins (VfuA00671, VfuA00673, VfuA00676, VfuA00680–VfuA00681, VfuA00684, and VfuA00689–VfuA00690). Genes encoding the preprotein translocase (SecY) subunit and the DNA-directed RNA polymerase subunit alpha (RpoA; VfuA00691) are also located in R2.

Region R3 (44,369 bp—46,297 bp; VfuB00044) also comprises highly conserved genes throughout, and bears a threonyl-tRNA synthetase (VfuB00041), which is located on different chromosomes within the selected *Vibrio* (see above).

Region R6 (1,347,108 bp to 1,590,549 bp; VfuA01296–VfuA01532) encompasses genes for flagellar biosynthesis. Several genes encoding the sidophore, vibriobactin, are present.

Region R9 (1,031,067 bp—1,058,755 bp; VfuB00991—VfuB01011) has the most homology to other sequenced *Vibrio* and *P. profundum*. All CDS in this region are found in the compared *Vibrio* genomes, although not all CDS contain regions that share at least 100 bp with 80% homology to the *V. furnissii* sequence. Those that do, however, include genes encoding 4 subunits of cytochrome O-ubiquinol oxidase (VfuB01000, VfuB01001, VfuB01002, VfuB01003) and protoheme IX farnesyltransferase (VfuB01005).

Region R10 spans from VfuB01141 to VfuB01201 (1,200,901 bp—1,265,108 bp) and includes 17 hypothetical proteins (VfuB01147, VfuB01172, VfuB01173, VfuB01176, VfuB01177, VfuB01178, VfuB01179, VfuB01180, VfuB01181, VfuB01182, VfuB01183, VfuB01184, VfuB01187, VfuB01188, VfuB01190, VfuB01192 and VfuB01193), two ABC transporters (VfuB01195; VfuB01196; VfuB01197; VfuB01198 and VfuB01154; VfuB01155; VfuB01156; VfuB01158), an operon repressor (VfuB01200), a transcriptional regulator (VfuB01201) for ribose metabolism, 3 transcription factors and regulators (VfuB01145, VfuB01149, VfuB01159, VfuB01165, VfuB01169, VfuB01171, VfuB01186). Bioinformatic analyses show that one of the ABC transporters is involved in ribose transport, whereas for the second the substrate needs to be identified.

In addition to the CDS in these regions, the *V. furnissii* genome has 126 other sequences with over 80% nucleotide homology that are represented in all the sequenced genomes used in this investigation. For example, the gene encoding glyceraldehyde-3-phosphate dehydrogenase (VfuA02488) is found in all analyzed strains (Figure 1, highlighted by an arrow).

### Regions unique to *Vibrio furnissii*

Regions R4 (101,225 bp— 169,686 bp; VfuA00089–VfuA00153) and R5 (2,125,032 bp—2,226,901 bp; VfuA02027–VfuA02133) are unique to the *V. furnissii* genome, and show no nucleotide homology over 80% with the other, sequenced *Vibrio* genomes.

Genes in region R4 encode peptides with highly conserved primary sequence domains and possess peptide homologs throughout the *Vibrio*. For example, BLAST analysis of the protein sequence of VfuA00116, which encodes UDP-glucose 6-dehydrogenase, revealed the closest related species to be *P. profundum* SS9. However, the nucleotide composition in R4 is markedly different from the rest of chromosome I, with a G+C content of 43.9% compared to 50.73% (Figure 1, track 14).

Region R5 contains a cluster of 13 genes encoding a hydrogenase 4 (VfuA02044–VfuA02062), a putative operon that encodes formate dehydrogenase (VfuA02050–VfuA02054 and VfuA02056) and a sequence encoding a beta-galactosidase (VfuA02089), which are not present in the other *Vibrio*. The majority of these genes show homology to *Aeromonas salmonicida* (Reith et al., 2008). This finding is supported by the phylogeny of these proteins (**Figure 4**) and may have risen through HGT. In addition to these unique sequences, it also contains genes encoding maltose ABC transporter-associated proteins (VfuA02090–VfuA02094), two tRNAs for Valine (VfuAtRNA63, VfuAtRNA64), a seryl-tRNA synthetase (VfuA02064), a *Lac*I-family transcriptional regulator (VfuA002095), proteins associated with vitamin B12 synthesis (VfuA02105–VfuA02109) and a galactoside O-acetyltransferase (VfuA02086), that have homologs in other *Vibrio* but with a nucleotide identity below 80%.

Region R7 (2,591,121 bp—2,637,073 bp; VfuA02488–VfuA02545) represents another unique region in the *V. furnissii* genome. However, in contrast to R4 and R5 is characterized by sequences derived from prophage CTX, and is the only significant phage footprint identified in the *V. furnissii* genome.

One noticeable feature of the *V. furnissii* genome is the presence of one single, large CDS encoding a polypeptide containing 3150 amino acids and with a predicted molecular mass of 329.18 kDa (Region R8 (345,688 bp—355,137 bp; VfuB00340)). The protein is also predicted to contain one signal peptide domain with no trans-membrane helix and 16 VCBS domains (Hemolysin-type calcium-binding repeat) (Baumann et al., 1993). VCBS domains are features of the RTX toxins, a diverse group of pore-forming exotoxins that are synthesized by many gram-negative bacteria and include cytolytic toxins, metalloproteases and lipases (Rodkhum et al., 2006; Frans et al., 2011; Satchell, 2011).

### *Vibrio furnissii* phylogeny

**Figure 2:**
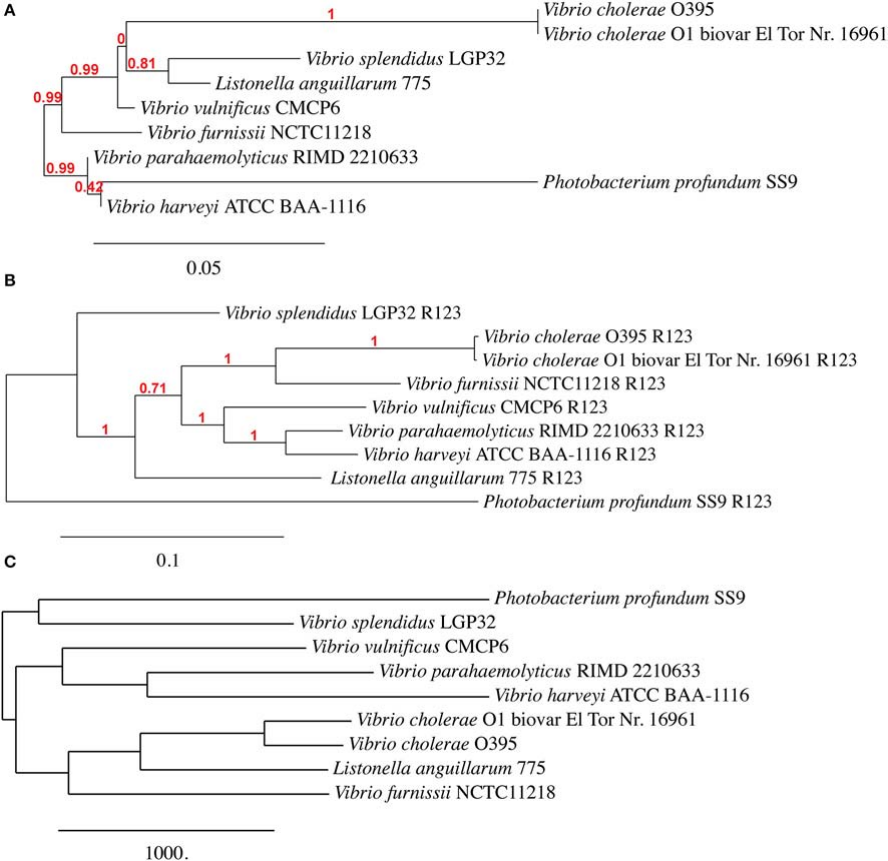
**Phylogenetic trees based on (A) 16S rRNA, (B) from concatenated nucleotide sequences from regions R1, R2 and R3 and (C) based on *Vibrio* pangenome from *V. furnissii* NCTC 11218, *V. cholerae* O1 biovar El Tor Nr. 16961, *V. cholerae* O395, *V. parahaemolyticus* RIMD 2210633, *V. vulnificus* CMCP6, *V. harveyi* ATCC BAA-1116, *V. splendidus* LGP32, *L. anguillarum* (formerly *V. anguillarum*) 775 and *P. profundum* SS9**.

Given the level of observed homology between the emergent *Vibrio*, the sequences present in R1 and R2 (Chromosome I), and R3 (Chromosome II) are ideal candidates for confirming phylogenetic relationships that have been established using 16S rRNA homology. Although R9 shows a high homology between the selected genomes, this region was not included because the concatenated nucleotide sequence failed to build a phylogenic tree. We therefore constructed a phylogenetic tree using all of the sequence information in regions R1, R2 and R3 (a total of 36 sequences) and constructed a dendogram using the *Vibrionaceae* 16S rDNA sequences (9 sequences). Additionally, we constructed a phylogenetic tree based on a pangenome analysis including both chromosome sequences of 9 *Vibrio* strains. Comparison between the three trees (Figure 2) revealed comparable phylogenetic relationships between *P. profundum, V. parahaemolyticus, V. harveyi* and *V. vulnificus*. However, the positions of *V. splendidus* and *Listonella anguillarum* vary more in each of the three trees (Figure 2). Most significantly, our analysis suggests a closer grouping between *V. furnissii* and the two *V. cholerae* strains than when 16S rRNA sequences, alone, are used.

A systematic analysis of the phylogeny of each *V. furnissii* CDS was performed to determine their possible function and identify those sequences that are uniquely represented in *V. furnissii*. To illustrate this analysis, the number of bacterial species in the NCBI database with sequences homologous to that of the interrogating *V. furnissii* CDS was plotted against the number of CDS with an equal amount of species. For further characterisation the CDS were matched to COG categories.

This distribution of phylogenetic trees can be divided into 4 ranges: 3–10 species per branch, 11–100 species per branch, 101–1000 species per branch and more than 1001 to species per branch (Figure 1, track 5: Red: 3–10; Green: 11 to; Blue: 101 to; Cyan: more than 1000).

353 CDS of chromosome I and 195 of chromosome II fall into the lowest category (from 3 to 10). The minimum of 3 was chosen, to ensure the generation of meaningful trees. Most proteins can be categorized as hypothetical and flagellar related. 14.7% (Chromosome II: 2.6%) of the proteins in this group belong to metabolism, 0.8% (Chromosome II: 0%) to cellular processes and signaling and 5.9% (Chromosome II: 23.6%) are grouped to information storage and processing. 78.6% (Chromosome II: 73.8%) have not been assigned a COG category.

The second, or lower mid-range (from 11 to 100) category contains 638 CDS of chromosome I and 412 of chromosome II. These are mainly hypothetical proteins. On Chromosome I 30.0% (Chromosome II: 15.5%) of the proteins within this range are in the metabolism COG set, 3.0% (Chromosome II: 0.7%) are cellular processes and signaling and 12.5% (Chromosome II: 28.2%) are for information storage and processing. Again, the majority (Chromosome I: 54.5%/Chromosome II: 55.6%) are not categorized.

The range from 101 to 1000 contains 1491 CDS from chromosome I and 511 CDS from chromosome II. Amongst these are ribosomal sequences that are classified as several tRNA synthetases. COG set metabolism makes up to 68.6% (25.2%), 2.3% (1.1%) are cellular processes and signaling related and 9.3% (41.0%) are information storage and processing. Only 19.8% have not assigned a COG term, contrasting with 32.7% on chromosome II.

The last category is composed mainly of proteins within the COG sets metabolism (80%/17.7%), cellular processes, and signaling 0.8% (0%) and metabolism 10.4% (55.8%), such as ABC-Transporters or proteins involved in fatty acid and cell wall biosynthesis. Not categorized are only 8.8% (26.5%) of the proteins.

### The super integron of the *V. furnissii*

**Figure 3:**
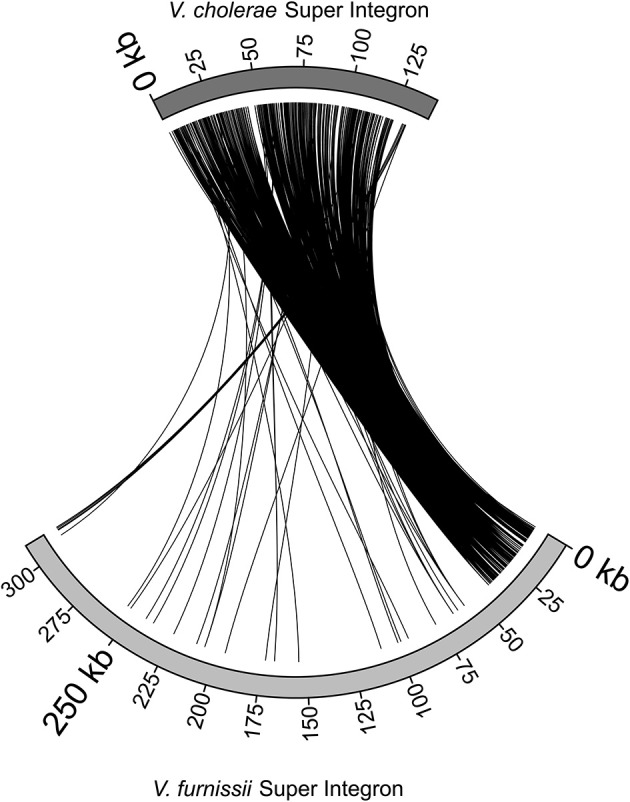
**Comparison of the Super Integrons of *V. cholerae* O1 biovar El Tor Nr. 16961 and *V. furnissii* NCTC11218**.

The Super Integron (SI) is a common feature amongst the *Vibrio* species, but it is also highly variable. The Super Integron of *Vibrio furnissii* is situated on chromosome II and encompasses the CDS VfuB01511 and VfuB00281 (1,591,485–281,565) stretching over a total of 311,971 bp. This is largest SI identified in any *Vibrio* strain. The *V. furnissii* SI starts with site-specific recombinase IntIA (VfuB01511) and terminates at VfuB00281, an anaerobic ribonucleoside-triphosphate reductase. The SI also encompasses 3 *att*C sites, which are a characteristic feature of Super Integrons. A third of the CDS within the SI are annotated as hypothetical, but many could be ascribed a function. These are mainly ABC transporters. A more detailed comparison between the *V. furnissii* and the *V. cholerae* SIs shows a high degree of conservation between both elements (Figure 3). However, it is clearly visible by the disruption at the end of the SI that a high amount of genomic rearrangements occurred in this region.

### Pathogenic and virulence related features of the *V. furnissii* genome

Based on established studies, we scanned the *V. furnissii* sequence for virulence-related features including quorum sensing, biofilm formation, virulence factors, chitin metabolism and natural competence.

Many bacterial species communicate by the secretion and detection of small signaling molecules called autoinducers. In *V. furnissii* two different types of quorum sensing mechanisms are present. The CAI-1 autoinducer, a substituted 13-carbon alkane, is produced by VfuB00269 (CqsA) and detected by VfuB00270 (CqsS), both located on chromosome II. The second autoinducer AI-2, a furanosyl borate, is produced by VfuA02965 (LuxS) on chromosome I and detected by VfuB00359, VfuB00358 (LuxP, LuxQ) on chromosome II.

In addition LuxU (VfuA02581) is phosphorylated by the unbound sensor kinases CqsS and LuxP/Q, which in turn phosphrylates LuxO (VfuA2580). The genes encoding LuxU/O are located on chromosome I in *V. furnissii*.

The *lux*-Operon is reported to be closely linked with HapR (VfuA00883), which controls biofilm formation by repressing the *vps*T transcription factor (VfuB00730) and controlling expression of genes for products that alter intracellular c-diGMP second messenger molecules (Tsou et al., 2009). Based on the *V. cholerae* QS model, biofilm formation requires Qrr-dependent repression of *hap*R translation to permit expression of the *vps*L-N exopolysacharide biosynthesis genes (Bardill et al., 2011). HapR is a TetR family transcriptional regulator that has been shown to regulate a variety of phenotypes important for both virulence and environmental survival (Tsou et al., 2011). VpsT is located on chromosome II of *V. furnissii*.

The major pilin subunit *tcp*A (VfuA00872) is present in *V. furnissii*. Being part of the toxin co-regulated pilus (TCP), it has been reported as a critical colonization factor (Manning, 1997).

*V. furnissii* has been reported to be a chitinovorous marine bacterium (Yu et al., 1987), (Bassler et al., 1991a,b; Yu et al., 1991) and there is bioinformatic evidence of at least one extracellular chitinase ChiA-2 (VfuB01027). Furthermore, a chitinase is encoded by *vfuA02755* and *vfuB00330* encodes for a chitodextrinase. The N-acetylglucosamine-binding protein A is also present in *V. furnissii* (VfuB01209).

### Genomic and pathogenicity islands of the *V. furnissii* genome

The presence of genomic islands in the sequenced *Vibrio* was determined using IslandPick, SIGI-HMM and IslandPath-DIMOB (summarized in Table 1).

**Table 1:** **Number of identified GEI in *V. furnissii* NCTC 11218, *V. cholerae* O1 biovar El Tor Nr. 16961, *V. cholerae* O395, *V. parahaemolyticus* RIMD 2210633, *V. vulnificus* CMCP6, *V. harveyi* ATCC BAA-1116, *V. splendidus* LGP32, *L. anguillarum* 775 and *P. profundum* SS9**.

**Chromosome**	**Identification method**					
	**SIGI-HMM**		**Islandpath-DIMOB**		**IslandPick**	
	**I**	**II**	**I**	**II**	**I**	**II**
*V. furnissii*	6	5	4	–	–	–
*V. cholerae*	11	5	2	3	–	–
*V. cholerae* O395	9	6	3	4	–	–
*V. parahaemolyticus*	16	6	5	3	5	12
*V. vulnificus*	17	5	4	3	4	–
*V. splendidus*	24	10	4	2	–	–
*V. harveyi*	30	2	15	10	9	–
*L. anguillarum*	19	8	7	2	–	–
*P. profundum*	12	17	7	4	–	–

In all *Vibrio* species analyzed, GEIs could be identified by the SIGI-HMM and/or the Islandpath-DIMOB methods. IslandPick could only identify GEIs in *V. parahaemolyticus*, *V. vulnificus* and *V. harveyi*. The highest number of GEIs was identified using SIGI-HMM software. The highest number of proteins within identified GEIs is in *V. harveyi* chromosome I (434), the lowest in *V. furnissii* and *V. cholerae* (both 114). On chromosome II the highest number of proteins is in *V. parahaemolyticus* (355), the lowest in *V. furnissii* (19). Most proteins within these GEIs are annotated as hypothetical proteins, transposases or ribosomal proteins and are present in all *Vibrio*. Interestingly, region R5 and R6 cover many CDS that are part of the predicted GEIs. The phylogenetic trees of these proteins show the variability and conservation of these regions between the different *Vibrios*. For instance VfuA01497, a transcriptional regulator belonging to the TetR family, (**Figure 5F**) is only present in the two *V. cholerae* strains, whereas VfuA01991, a E1-E2 family cation transport ATPase, seems to be closely related to *V. harveyi* and *V. parahaemolyticus* (**Figure 5E**). Similar evidence for HGT can be found throughout all predicted GEIs.

## Discussion

Our analysis of the distribution of open reading frames of the *V. furnissii* genome emphasizes two aspects of *Vibrio* genetics. First, the majority of the predicted coding sequences with over 80% similarity to other genes is located on chromosome I. Second, the proportion of CDS that display 60% or less identity to previously identified genes are predominantly found on chromosome II. This distribution is consistent with previous observations that the larger of the two chromosomes in *Vibrio* predominantly contains genes encoding functions corresponding to growth and survival under standard conditions, such as housekeeping genes, which are less likely to be divergent (Dryselius et al., 2007). In other *Vibrio* species, genes that encode functions beneficial for unusual growth situations tend to be located on the smaller chromosome (Jain et al., 2002; Philippe and Douady, 2003; Dryselius et al., 2007). Also there is a high representation of COGs in the “metabolism” set compared to other *Vibrio* which may indicate that *V. furnissii* has a greater than expected capacity to adapt to, and exploit, diverse environmental niches. Additionally the CDS present on chromosome II tend to be shorter and more often disrupted by random nucleotide changes than the CDS present on chromosome I, which is compounded by a highly variable G+C content on chromosome II (Figure 1, track 1/2—red group; track 14). Possible explanations for this difference in gene organization between the *V. furnissii* chromosomes include a lesser selective pressure on chromosome II and/or a lesser need for repair which, taken together, would result in an increased possibility of retained mutations on chromosome II, generating novel genes (Garcia-Vallve et al., 2000; Dryselius et al., 2007). It is also possible that, under certain conditions, differences in the copy number of predominantly “housekeeping” chromosome I and “variable” chromosome II might occur, potentially increasing the effective level of expression of genes on chromosome II, to the bacterium's advantage (Heidelberg et al., 2000; Schoolnik and Yildiz, 2000; Jain et al., 2002; Philippe and Douady, 2003; Dryselius et al., 2007). Also, the G+C content of the *Vibrio furnissii* genome is 4–10% higher than other sequenced *Vibrio* species, possibly indicating higher levels of HGT (Garcia-Vallve et al., 2000) in *V. furnissii* than in the other, sequenced *Vibrio*. This is also supported by the position of the predicted GEIs, which fall in regions with a change in G+C contents (Figure 1, track 14).

It is well established that proteins or enzymes that interact directly with DNA are under high selection pressure, and therefore it is not surprising that the homology between these genes is so high across the sequenced genomes. This level of conservation exists across the entire bacterial phylogeny, as evidenced by the fact that all genes in R1 and R2 have 101–1000 sequences in their respective phylogenetic trees (Figure 1, track 5— blue group) except for VfuA00671 which encodes the 30S ribosomal protein S19, and has only 11–100 homologous sequences in the phylogeny (Figure 1, track 5—blue group).

**Figure 4:**
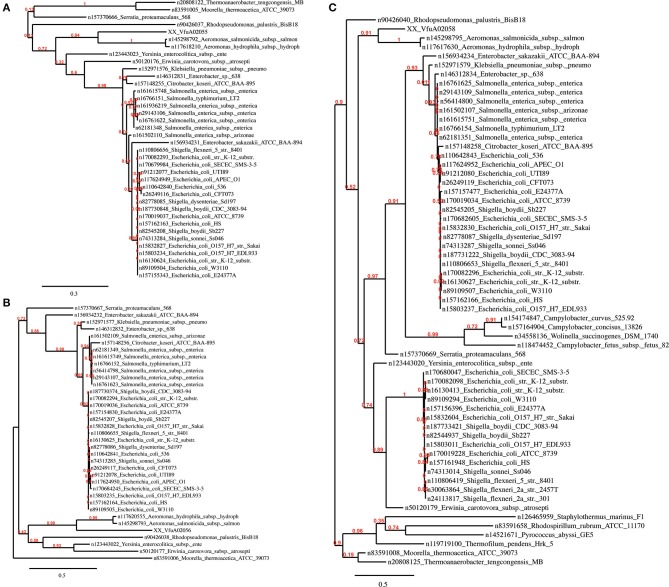
**Phylogenetic trees of the *V. furnissii* proteins (A) VfuA02055, (B) VfuA02056, and (C) VfuA02058 showing their homology to *Aeromonas salmonicida***. Numbers representing branch support values.

It is plausible that the divergence of R4 arose through the accumulation of silent mutations in an unusually A+T rich portion of the *V. furnissii* genome. Novel features of *V. furnissii* are clearly the presence of a hydrogenase 4 and formate dehydrogenase (see Region R5), which have high homology to *Aeromonas salmonicida* (Figure 4), although their functions in *V. furnissii* remain unclear. Consequently, R5 contains suites of genes that are unique to *V. furnissii* and probably arose via HGT, interspersed with sequences that have homologs (albeit poor homologs) within the other *Vibrio*. Interestingly genomic islands have been predicted in region 5. These are fundamental differences between the sequences in R4 and R5 that are suggestive of different evolutionary histories between these regions.

Our analyses of the phylogeny of the selected 9 *Vibrio* strains reveals that the position of *V. splendidus* and *Listonella anguillarum* within a phylogenetic tree is dependent on the method used to construct the phylogenetic tree. This observation can possibly be explained by variations within the smaller chromosome, although more work must be done to support this hypothesis. A second observation is that *V. furnissii* groups more firmly within the *V. cholerae* group than a phylogenic analysis based only on 16S rRNA sequences suggests.

The super integron common in *Vibrio* is also present in *V. furnissii*. Although the *V. furnissii* super integron still shows characteristic features, such as the *att*C sites, it has an increased size and many genomic rearrangements. This underlines the high genomic variability of the SI region.

Amongst the novel features shown by our analysis is VfuB00340 located on chromosome II, which is likely to belong to the RTX protein family. Homologs to putative RTX toxins have been identified in *V. vulnificus* and may play a role in cell adhesion and/or pathogenesis. In *V. anguillarum*, RTX toxins are a major component of virulence. A phylogenetic analysis of the amino acid sequence of VfuB00340 failed to return any homologous sequences. However, the presence of calcium binding sites and von Willebrand factor type A, and repetitive VCBS domains indicates that VfuB00340 may be a novel toxin of the RTX protein family, although further *in vivo* analysis is required to confirm its precise function. One of the supposed functions of the RTX protein is be to support haemolysis. The major virulence factor of *Vibrios*, haemolysin, encoded by the gene *hyl*A is present in *V. furnissii* (VfuB01125) and located on chromosome II (Li et al., 2008) demonstrated contribution of RTX toxins to haemolytic activity in *L. anguillarum*. Although a *rtx* gene cluster is not present as such, *V. furnissii* possesses upstream of VfuB0340 several genes that could be involved in this novel toxic activity (VfuB0341–VfuB0347), such as two RTX toxin transproters (VfuB00343 and VfuB00346). However, the sequence encoding the toxin secretion transporter is missing from the *V. furnissii* genome, although it is quite possible that its function is performed by another secretion protein.

There have been reports linking *Vibrio* motility to virulence, with motility being down-regulated and virulence factor expression simultaneously up-regulated. Silva et al., observed that transcription of *tox*T, *ctx*A, and *tcp*A is up-regulated in a *V. cholerae* non-motile (*mot*Y) strain (Silva et al., 2006). Supporting evidence for this model comes from Ghosh et al., who found that the histone-like nucleoid structuring protein H-NS stimulates motility by stimulating *flr*A expression while repressing *ctx*AB and *tcp*A transcription (Ghosh et al., 2006).

In response to the appropriate environmental signal(s), tcpA is stimulated by TcpI, while simultaneously reducing *Vibrio* chemotaxis-directed motility (Harkey et al., 1994). In contrast to *V. cholerae*, *tcp*I is located on the second chromosome of *V. furnissii* (VfuB00809). TcpI was identified as a ToxR-activated gene that encodes an inner membrane protein with extensive sequence similarity to the highly conserved signaling domain in methyl-accepting membrane chemoreceptors and plays an important role in colonization of the small bowel. Both *V. cholerae* regulatory proteins ToxR and ToxT, which are involved in activation of various virulence genes, such as the above-mentioned CT (Miller et al., 1987; Krukonis et al., 2000) or the expression of OmpU (VfuA00928) that mediates resistance to bile and anionic detergents (Mathur and Waldor, 2004), are also absent in *V. furnissii*. However, the regulatory protein ToxS, a protein known to interact with ToxR and stimulate ToxR activity, is present in *V. furnissii* (VfuA02617) (DiRita and Mekalanos, 1991). This observation suggests the presence of an unknown protein carrying out a function similar to *Vc*ToxR, in *V. furnissii*.

Bioluminescence, which has been well characterized in *V. harveyi*, is missing in *V. furnissii*. However, some genes belonging to the lux family and related quorum sensing systems are present. These genes are closely linked to the *Vibrio* competence system. This competence system is also dependent on chitin utilization. Chitin, composed of 1,4-linked GlcNAc residues, induces the expression of a 41-gene regulon in *V. furnissii* that is involved in chitin colonization, digestion, transport, and assimilation, and includes genes predicted to encode a type IV pilus assembly complex (Meibom et al., 2004, 2005). The presence of several chitinase and associated genes in *V. furnissii* confirms previous reports and suggests *V. furnissii* to be naturally competent. However, two additional chitinases found in *V. cholerae* are lacking in *V. furnissii*. In the presence of chitin, TfoX (VfuA01621) is induced (Yamamoto et al., 2011) and is thought to play a role in controlling the transcription of *com*EA (Bardill et al., 2011). The *com*-system is essential for natural competence (i.e., cells' ability to uptake DNA from the environment) and incorporation of extracellular DNA into the genome which is one of the driving motors for HGT and the generation of genomic islands (Scrudato and Blokesch, 2012).

Bacterial genomes typically harbor a variable number of accessory genes acquired by HGT that encode adaptive traits that are beneficial to the host under certain growth or environmental conditions (Schmidt and Hensel, 2004). These so called Genomic Islands (GEIs) are clusters of genes that are typically recognized as discrete DNA segments between closely related bacterial strains. It is widely recognized that HGT has played a crucial role in the evolution of bacterial species. Furthermore, several lines of evidence suggest the existence of evolutionary ancient GEIs spread over versatile groups of otherwise unrelated bacteria (Juhas et al., 2009). Evidence therefore suggests that the formation of GEIs contributes to the diversification and adaptation of microorganisms, and the presence of GEIs may confer a significant impact on genome plasticity and evolution, the dissemination of antibiotic resistance and virulence genes, and the formation of catabolic pathways (Juhas et al., 2009). The sequences of GEIs display certain properties that mark them as being atypical compared to the overall genome of the organism in which they are found. These features include a large chromosomal region present in a subset of isolates of a given species and absent from other isolates of the same species; the presence of loci involved in genomic mobility such as integrases and transposases; the association with one or more tRNA genes; the presence of flanking direct-repeat sequences that mark the site where incoming DNA recombined with the host genome; a G+C content that differs significantly from that of the host organism; and an instability in chromosomal insertion sites. The formation of GEIs is still poorly understood, although at least one of three primary mechanisms, conjugation, transduction, and transformation, is believed to be involved (Juhas et al., 2009). GEIs are classified based on the different functions they encode, which include metabolic islands, degradation islands, resistance islands, symbiosis islands, and pathogenicity islands (Murphy and Boyd, 2008).

**Figure 5:**
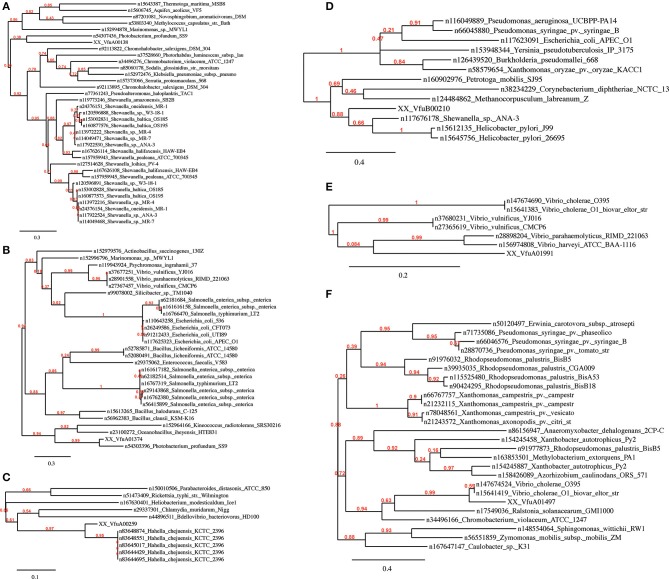
**Phylogenetic trees of the *V. furnissii* proteins (A) VfuA00138, (B) VfuA01374, (C) VfuA00259, (D) VfuB00210, (E) VfuA01991, and (F) VfuA01497 showing putative horizontal gene transfer events**. Numbers representing branch support values.

As the nomenclature suggests, pathogenicity islands (PAIs) are unstable chromosomal regions that bear virulence-related genes and are therefore associated with different virulence-associated characteristics and phenotypes. The PAIs that arose through HGT generally exhibit a G+C content that diverges from that of the host genome, a gene or genes encoding a P4-like integrase, and a chromosomal insertion at a tRNA-serine locus that is flanked by direct repeats (Groisman and Ochman, 1996; Dobrindt et al., 2004). Out of the 114 proteins within the identified GEIs on *V. furnissii* genome, only 63 (see supplement) could be allocated with phylogenetic trees. The proteins with no tree are mainly hypothetical proteins, which typically have no or a low branched tree. One other possibility is that there are too many species involved in tree building, which will also cause the pipeline program to fail. For instance, one hypothetical protein (VfuA00164) has a higher homology to *V. harveyi*, *V. splendidus*, *L. anguillarum*, and *P. profundum* than to *V. cholerae*. These 4 species are marine bacteria and the protein may therefore have a function that is pertinent to the marine pathogens. One other particularly interesting protein is VfuB00210 (Figure 5D), only present in *V. furnissii*, also a hypothetical protein with a conserved domain of unknown function (COG4694). Further determination is therefore required to ascribe functional reference to theses identified sequences.

Evidence for horizontal gene transfer not only comes from the identification of GEIs in the *V. furnissii* genome, but can be also found in the phylogenic trees. The protein VfuA00259 (a cell wall-associated hydrolase) is deduced to be related to *Hahella chejuensis*, a marine proteobacterium (Figure 5C). As already stated above, *Aeromonas salmonicida* may have been the donor for various transfer events (Figure 4). The phylogenetic trees also suggest gene transfer between more closely related strains such as *P. profundum* and *V. furnissii* (Figures 5A,B). These results suggest a high frequency of HGT between *V. furnissii* and bacteria that occupy a similar ecological niche.

Even if none of the major pathogenicity islands are present in *V. furnissii*, it cannot be ruled out that *V. furnissii* bears a pathogenic potential for humans and, more immediately, to marine arthropods which are the basis of marine economic activity in regions that are already vulnerable to climate change. The presence of a novel RTX type protein is clear evidence of this potential. Furthermore, the appearance of hydrogenase 4 and formate dehydrogenase, which originated from non-closely related bacteria, alongside functional QS and competence systems shows a high potential to assimilate virulence related genes through HGT and therefore quickly increase the pathogenicity of *V. furnissii*.

### Conflict of interest statement

The authors declare that the research was conducted in the absence of any commercial or financial relationships that could be construed as a potential conflict of interest.
